# Single Word Intelligibility of Individuals with Parkinson’s Disease in Noise: Pre-Specified Secondary Outcome Variables from a Randomized Control Trial (RCT) Comparing Two Intensive Speech Treatments (LSVT LOUD vs. LSVT ARTIC)

**DOI:** 10.3390/brainsci11070857

**Published:** 2021-06-27

**Authors:** Geralyn Schulz, Angela Halpern, Jennifer Spielman, Lorraine Ramig, Ira Panzer, Alan Sharpley, Katherine Freeman

**Affiliations:** 1 Department of Speech, Language, and Hearing Sciences, College of Arts and Sciences, George Washington University, Washington, DC 20052, USA; 2 LSVT Global, Inc., Tucson, AZ 85705, USA; Angela.Halpern@lsvtglobal.com (A.H.); jennifer@frontrangevoicecare.com (J.S.); ramig@colorado.edu (L.R.); 3 National Center for Voice and Speech-Denver, Denver, CO 80204, USA; 4 Department of Communication Sciences and Disorders, Teachers College, Columbia University, New York, NY 10027, USA; 5 Department of Speech, Language, and Hearing Sciences, College of Arts and Sciences, University of Colorado—Boulder, Boulder, CO 80309, USA; 6 Dynastat, Inc., Austin, TX 78731, USA; ilpanzer@dynastat.com (I.P.); asharpley@dynastat.com (A.S.); 7 Department of Biomedical Sciences, Charles E. Schmidt College, Florida Atlantic University, Boca Raton, FL 33431, USA; kfreemancostin@health.fau.edu

**Keywords:** dysarthria, motor speech disorders, prosody treatment, LSVT LOUD, Parkinson’s disease

## Abstract

The majority of people with Parkinson’s disease (PD) experience both prosodic changes (reduced vocal volume, reduced pitch range) and articulatory changes (imprecise articulation) that often limit speech intelligibility and may contribute to significant declines in quality of life. We conducted a randomized control trial comparing two intensive treatments, voice (LSVT LOUD) or articulation (LSVT ARTIC) to assess single word intelligibility in the presence of background noise (babble and mall). Participants (64 PD and 20 Healthy) read words from the diagnostic rhyme test (DRT), an ANSI Standard for measuring intelligibility of speech, before and after one month (treatment or no treatment). Teams of trained listeners blindly rated the data. Speech intelligibility of words in the presence of both noise conditions improved in PD participants who had LSVT LOUD compared to the groups that had LSVT ARTIC or no treatment. Intensive speech treatment targeting prominent prosodic variables in LSVT LOUD had a positive effect on speech intelligibility at the single word level in PD.

## 1. Introduction

It has been estimated that approximately 6.1 million (range of 5.0–7.3 in 2016) people are living with Parkinson’s disease (PD) globally [1,2]. As many as 90% have speech signs that often reduce their ability to be understood and negatively impact their quality of life [3,4,5,6]. The hypokinetic dysarthria^.^ in PD may not be as immediately visible as some of the major limb motor signs (e.g., tremor); however, it affects all motor speech subsystems. The two most salient speech signs include disorders of prosody (reduced vocal loudness; monopitch, and monoloudness; a breathy or harsh voice) [6,7,8,9,10,11,12,13], and disorders of articulation (imprecision of consonant and vowel production) [6,14,15,16,17,18,19,20,21,22,23,24,25].

Prosodic disorders, specifically reduced vocal loudness (a prominent prosodic feature), is often the first speech sign noticed in people with PD [6,8]; voice is described as weak, hoarse, and breathy [26,27,28]. Early views associated reductions in vocal loudness with the rigidity and stiffness in the laryngeal and ribcage muscles and hypokinesia (reduced amplitude of movement caused by underlying dopaminergic deficiency) [28,29,30,31,32,33,34]. More recent studies have demonstrated abnormalities in central sensory processing (reduced awareness of soft voice), internal cueing (difficulty self-generating increased loudness), and self-monitoring of speech output that may play a causal role in the reduced vocal loudness of PD [35,36,37,38,39,40,41,42,43,44,45].

Articulation disorders are not uncommon in people with PD; Logemann and colleagues [4,16] found that articulation disorders occurred in 45% of the 200 PD patients they tested. Similarly, Ho et al. [8] found articulation impairment in 38.5% of the 200 they tested and that articulatory impairment increased in frequency as speech was more severely affected. The articulation disorder in PD has been described perceptually by characteristics including “imprecise consonants”, “prolonged phonemes”, “irregular articulatory breakdown”, and “distorted vowels” [26,27,28]. Of these perceptual characteristics, Darley and colleagues found that “imprecise consonants” was the most deviant perceptual characteristic of the articulation disorder in PD [26,27]. While the characterization of “imprecise consonants” may be clinically useful, it does not in fact describe the physiologic dysfunction occurring in the vocal tract that produces the perception of “imprecise consonants” [4]. Production of consonants differ from production of vowels in that consonant production requires that the vocal tract be constricted at some point (e.g., for a “p” there is total constriction at the lips; for a “f” the lower lip rises to the top teeth and produces a partial but tight constriction, etc.) whereas vowel production involves very little, if any, vocal tract constriction (e.g., for “a” the jaw remains open and the tongue lies flat on the floor of the jaw). Therefore, “imprecise consonants” implies a dysfunction of vocal tract constriction. Logemann and colleagues [4,16] found that consonants that require greater constriction in the vocal tract are the ones most misarticulated by people with PD (e.g., “p” or “f” vs. “r” or “h”).

These speech changes in prosody (loudness) and articulation have been reported to lead to significant declines in functional communication, communicative participation, and quality of life [6,22,46,47,48,49,50,51,52,53,54,55]. Neither medical (neuropharmacological or neurosurgical) nor traditional speech treatments, which focus on a range of motor speech symptoms (e.g., respiration, articulation, speech rate, loudness, intonation) in a low dosage mode, have proved consistently or significantly beneficial in improving the degenerative speech or intelligibility of people with PD [56,57,58,59]. This is likely due to the treatment focusing on only the motor speech symptoms in a low dose mode without regard to deficits in sensory feedback and internal cueing that have been associated with persistent speech disorders in PD [35].

An intensive speech treatment targeting voice, Lee Silverman Voice Treatment (LSVT LOUD), henceforth, intensive voice treatment, has produced the first evidence of short and long term (2-year) efficacy as demonstrated in three randomized control trials (RCT) [60,61,62]. Intensive voice treatment differs from traditional speech treatments in several ways. First, it focuses on a single treatment target of a prominent prosodic feature, vocal loudness, in contrast to providing instruction for multiple targets at once (respiration, articulation, speech rate, loudness, and intonation). For example, intensive voice treatment only instructs participants to speak louder and not, “Take a deep breath, speak louder and slower and raise your pitch”. Second, it follows principles of motor learning and promotes activity-dependent neuroplasticity [63] including intensive dosage (16 1-h sessions over one month) and high effort treatment. Third, it retrains sensory feedback and internal cueing. The focus on a single target LOUDNESS makes it feasible for the patients, often with cognitive and sensory challenges, to implement one treatment target in their functional communication and have a positive impact on multiple aspects of speech production with limited cognitive load. In addition to increased vocal loudness following intensive voice treatment, studies have documented other system wide effects such as improvements in intonation [64], aerodynamics [65], perceptual measures of voice quality [7], participant-reported communication effectiveness [62], as well as measures of vocal fold closure [66], swallowing [67,68], and facial expression [69,70]. Further, studies of neural changes (positron emission tomography and functional magnetic resonance imaging) following intensive voice treatment indicate effects that go well beyond vocal loudness [71,72,73].

### 1.1. Speech Intelligibility

Speech intelligibility, the extent to which others can understand speech, is of great functional significance and has been used to document severity of dysarthria [74] and the efficacy of treatment for dysarthria (e.g., Levy et al., [75]). The study of speech intelligibility in neurodegenerative disorders in general, and in PD in particular, is complicated by several factors. One factor is the specific type of speech sample that is used: single words, sentences, reading passages, and spontaneous speech. Each of these speech sample types provides different levels of information, and no single measure of intelligibility will serve every clinical or research purpose [76]. Indeed, the two most widely used dysarthria assessments, the Frenchay dysarthria assessment [77] and the assessment of intelligibility in dysarthric speakers [74] assess intelligibility at both the single word and sentence level. A sentence level measure of intelligibility will provide an overall measure of intelligibility but cannot provide details regarding why a speaker has that intelligibility deficit. This is because sentence level measures are not constructed to control several factors that contribute to variability in intelligibility scores [76]. Another way to look at this is that two speakers can have the same overall sentence intelligibility score but very different speech deficiencies contributing to those same intelligibility scores. [76]. One valid reason for utilizing words to assess intelligibility (and in particular the DRT words) is to arrive at a phonetic interpretation of impaired intelligibility [76].

In addition to the use of single words allowing a phonetic feature analysis of errors, single words also have the advantage of eliminating a number of other variables that can affect intelligibility, such as sentence level syntactic and prosodic variables. It should also be noted that the use of single words to assess intelligibility is a much less difficult task for dysarthric participants than sentence level productions. As such, if an intelligibility impairment is noted at the single word level, intelligibility deficits are more than likely at “higher”/“more complicated” levels of speech productions, such as the sentence level [78,79,80].

Another factor that complicates the study of intelligibility is the listening environment in which intelligibility is measured, specifically, whether the stimuli are presented in a quiet listening environment or whether the stimuli are presented in the presence of background noise. Studies have investigated word intelligibility in quiet environments (i.e., without background noise). These studies have reported mixed results when simply cueing participants to increase loudness compared to healthy age matched controls [5,49,78,81]. Three of these studies reported significantly greater word intelligibility in HCs vs. PD participants [5,49,81], while one study reported no significant differences in word intelligibility between HCs and PD participants [78]. It should be noted that the Chiu and Forrest [49] study analyzed single words that were produced in a sentence context. It should also be noted that in all of these studies, the lowest intelligibility for single words was 83% for the participants with PD (range across studies of 83%—greater than 90%), while the lowest intelligibility for single words was 91% for the HC participants (range across studies of 91%—greater than 99%). When taken as a whole, these results indicate that while single word intelligibility for people with PD can be significantly reduced compared to HCs, single word intelligibility for people with PD is relatively good in quiet listening environments.

People with PD have difficulty maintaining intelligible speech in the presence of background noise [5,6,10,82,83,84]. Therefore, it is “ecologically” important to investigate the intelligibility of speech in people with PD in the presence of background noise. Fewer studies have investigated the intelligibility of words in noisy environments (i.e., in the presence of background noise). Both Chiu and Forrest [49] and Leszcz [81] found significant reductions in the intelligibility of words in participants with PD in the presence of background babble (multi-talker) noise, and this reduction in intelligibility was significantly greater in participants with PD (range of 44–69%) than in HC participants (range of 71–91%). These results confirm the difficulties that people with PD have being understood when speaking in noisy environments. The majority of studies that have assessed PD intelligibility in background noise have used multi-talker babble. In general, babble noise has been found to be more detrimental to speech perception than other types of background noise, such as mall noise [85]. This finding has been attributed to the differences in the spectral characteristics of background mall noise compared to background babble noise.

### 1.2. Loudness and Intelligibility

Another factor that affects intelligibility is the vocal loudness of the speaker. The vast majority of studies that investigated the impact of increased vocal intensity/loudness on speech intelligibility for people with PD, were conducted in quiet listening conditions and by simply cueing the person with PD to increase loudness [46,53,86,87]. In these studies, increased vocal loudness was found to increase intelligibility in PD participants for single words [86], phrases [46,87], sentences [86], and passages [53]. One study investigated the impact of increased vocal intensity/loudness on speech intelligibility for people with PD in noisy listening conditions and by simply cueing the person with PD to increase loudness [88]. In that study, PD participants read sentences in background babble noise; listeners rated their scaled intelligibility better in the cued loud condition compared to the habitual condition.

Several studies have investigated the impact of increased vocal intensity/loudness on speech intelligibility for people with PD in quiet listening conditions during un-cued speech tasks following intensive voice treatment [89,90,91]. In these studies, increased vocal loudness was found to increase intelligibility in PD participants for words extracted from read sentences [91], reading of passages [90], and conversational monologue [89]. Results from all three studies demonstrated increased intelligibility following intensive voice treatment. Two studies investigated the impact of increased vocal intensity/loudness on sentence intelligibility for people with PD in noisy listening conditions following LSVT LOUD treatment [75,92]. The Cannito et al. study [92] investigated orthographically-transcribed patients’ recorded sentences by unfamiliar listeners in background pink noise and found significant improvement in sentence intelligibility in their PD participants. In the only randomized controlled trial to date investigating sentence intelligibility in PD, Levy and colleagues [75] added background babble noise to the pre- and post-treatment sentence samples and found that PD participants in the intensive voice treatment group had significantly increased intelligibility compared to an articulation treatment group (an intensive treatment comparable to voice treatment, focusing on articulation) and untreated PD participants in the presence of background babble noise.

### 1.3. Articulation and Intelligibility

Perceptual studies have demonstrated that articulation has been the strongest contributor to speech intelligibility in motor speech disorders, including Parkinson’s dysarthria [93]. Acoustic studies suggest that speech intelligibility may be related to the extent of articulatory movement impairment in PD [94,95]. However, only three studies have examined this relationship. Forrest et al. [96] found smaller movement amplitudes and velocities for the jaw and lower lip in more affected PD speakers. Weismer et al. [97] demonstrated a positive relation between speed of tongue movement amplitude and velocity. Furthermore, they found a significant positive correlation between scaled intelligibility and average speed of the tongue but not the jaw or lips. Weismer concluded that measures of articulatory reduction, and specifically tongue motions, contribute to deficits in speech intelligibility in PD [97]. This finding is consistent with a more recent study [51], which also found a positive relation between movement amplitude of the tongue and scaled intelligibility in a sentence production task. Additionally, the Kearney et al. [51] results extend this relationship to the movement amplitude of the jaw. However, this study did not find that reduced tongue and jaw velocities were associated with lower ratings of intelligibility. Taken together, these studies all demonstrate that smaller amplitude movements of the articulators, particularly the tongue, play a significant role in the reduced intelligibility of PD speakers.

Several articulatory factors have been reported to account for the increase in intelligibility when people with hypokinetic dysarthria speak with increased intensity/loudness. A common finding across several studies is that when PD speakers are simply cued to increase vocal intensity/loudness there is an increase in the distinctiveness of vowel production [18,86,98,99,100] and consonant production [55,86]. Similarly, increases in the distinctiveness of vowel production have been found following intensive voice treatment [97,101,102,103]. Articulatory acoustic changes have also been reported following intensive voice treatment [103]. More specifically, Dromey et al. [103] found decreased mean frication duration following intensive voice treatment and associated it with more rapid glottal adjustments. These results suggest modifications in coordination of the glottal valving gesture with the oral constriction. Additionally, there were increases in second formant trajectory extent following intensive voice treatment. This observation was likely due to increases in jaw displacement accompanying louder speech (as demonstrated by Forrest et al. [96] and Kearney et al. [51]), which would allow more time for supraglottal articulator movement.

Articulation disorders occur frequently in PD and affect intelligibility. Like the Levy et al. study [75], we chose an intensive treatment that focuses on articulation (LSVT ARTIC), henceforth, intensive articulation treatment, to compare to the intensive voice treatment to determine which treatment has the greater effect on the intelligibility of single words.

### 1.4. Purpose

The purpose of this study was to determine whether increased loudness (targeting the prosodic system) or enhanced articulation (targeting the articulatory system) has the larger impact on improving the intelligibility of words in the speech of individuals with PD in noise. This design allows us to dissociate the specific contribution of the intensive dosage of treatment and the target of treatment by having two active treatment comparators. We used the DRT, an ANSI standard for measuring intelligibility in speech [104,105,106,107] in noise (mall and babble) and no-noise conditions to compare the impact of these two different intensive treatments on the intelligibility of words in people with PD compared to an untreated group of PD participants (UNTXPD) and a group of healthy controls (HCs). The DRT is comprised of 96 rhyming word pairs. The initial consonant of each word pair differs based on one of six distinctive phonetic features as defined by Jakobson, Fant, and Halle (compactness, graveness, sibilation, sustention, nasality, and voicing [108]; see Appendix A, Appendix B and Appendix C for further information on distinctive features and definitions of the distinctive feature categories). The DRT distinctive features capture the amount and place of constriction of English consonants, which makes the DRT an important tool for investigating vocal tract function during the production of consonants in single words.

### 1.5. Hypotheses

The following questions and resultant hypotheses were investigated in the present study:

1. Does intensive treatment targeting voice or targeting articulation increase vocal loudness in single words compared to untreated PD participants? To the best of our knowledge, this question has never been addressed. However, given that Ramig et al. [62] demonstrated an increase in SPL for sentence level material (reading passages, monologue) at 1 month post intensive voice and intensive articulation treatments, we hypothesized both treatment groups would demonstrate a significant increase in SPL for single words following treatment compared to the untreated group (UNTXPD). In addition, the Ramig et al. [62] results demonstrated that among groups, the intensive voice treatment group had significantly greater SPL than both the intensive articulation treatment and UNTXPD groups at 1 and 7 months post treatment. We, therefore, hypothesized that the intensive voice treatment group would demonstrate significantly greater SPL post treatment than both the intensive articulation treatment and UNTXPD groups.

2. What is the difference in single word intelligibility for PD participants pre-treatment compared to HCs?

2a. In quiet environments (i.e., no-noise condition), are HC participants more intelligible than PD participants? Three studies reported significantly greater word intelligibility in HCs vs. PD participants [5,49,81], while one study reported no significant differences in word intelligibility between HCs and PD participants [78]. Therefore, we hypothesized that HCs would be significantly more intelligible than the combined PD groups in the no-noise condition pre-treatment.

2b. In the presence of background noise (i.e., mall and babble noise conditions), are HC participants more intelligible than PD participants? People with PD have difficulty maintaining intelligible speech in the presence of background noise [5,6,10,82,83,84]. Chiu and Forrest [49] and Leszcz [81] found significant reductions in the intelligibility of words in participants with PD when compared to HCs in the presence of background babble noise. Therefore, we hypothesized that the HCs would be significantly more intelligible for single words than the combined PD groups in both background noise conditions.

3. What is the effect of treatment on word intelligibility in PD in the ecologically valid situation of background noise?

3a. In the presence of background noise (i.e., mall and babble noise conditions), are treated PD groups more intelligible than UNTXPD participants? Previous studies [75,92] have demonstrated increased sentence intelligibility in background noise for PD participants treated with intensive voice treatment. In addition, single word production is a relatively less difficult task compared to sentence production and eliminates other variables that affect intelligibility of sentences [76]. Therefore, we hypothesized that the intensive voice and intensive articulation groups would have significantly greater word intelligibility post-treatment compared to the UNTXPD group.

3b. In the presence of background noise (i.e., mall and babble noise conditions), is the intensive voice treatment group more intelligible than the intensive articulation and UNTXPD groups? Levy et al. [75] reported greater sentence intelligibility changes in the intensive voice treatment group compared to the intensive articulation group in the presence of background noise. Therefore, we hypothesized that the intensive voice treatment group would demonstrate greater single word intelligibility than the intensive articulation group in the presence of both background noise conditions following treatment.

4. What is the relationship between SPL and word intelligibility? Previous studies have demonstrated increased sentence intelligibility following intensive voice treatment [75,89,92]. Therefore, we hypothesized that as SPL increases, mean DRT scores would also increase, especially in the two noise conditions.

## 2. Materials and Methods

### 2.1. Trial Design

The study design is an unblinded RCT in PD participants comparing two behavioral speech treatments with different targets (voice or articulation) matched on intensive dosage relative to untreated PD controls. The data are single word intelligibility (diagnostic rhyme test (DRT)) and sound pressure level (SPL) and are considered pre-specified secondary outcome variables as they were collected as a part of the Ramig NIH-NIDCD R01 DC0115 randomized controlled trial (RCT). These data have never been published before. The initial publication on this RCT was Ramig et al., 2018 [62], which reported SPL in reading and spontaneous speech as the primary outcome variable, and the participant reported modified communication effectiveness index (CETI-M) as a secondary variable. Subjects from the Ramig et al., 2018 RCT [62] are subjects in this current Schulz paper and the Levy et al., 2020 [75] paper, which reported listeners’ orthographic transcription accuracy from spontaneous speech as the primary outcome variable.

The Ramig et al., 2001b [61] RCT studied voice treatment and respiratory treatment pre, post, 12, and 24 months follow-up, and the Ramig 2001a [60] RCT studied voice treatment and untreated PD and untreated healthy controls pre, post, and six months follow-up. In both 2001a,b, [60,61] RCTs, SPL was the primary outcome variable, with fundamental frequency variability (F0STSD) as a secondary variable in Ramig et al., 2001b [61]. These two 2001 studies [60,61] were independent data sets of subjects.

### 2.2. Participants

#### 2.2.1. Parkinson’s Disease Participants

**Figure 1:**
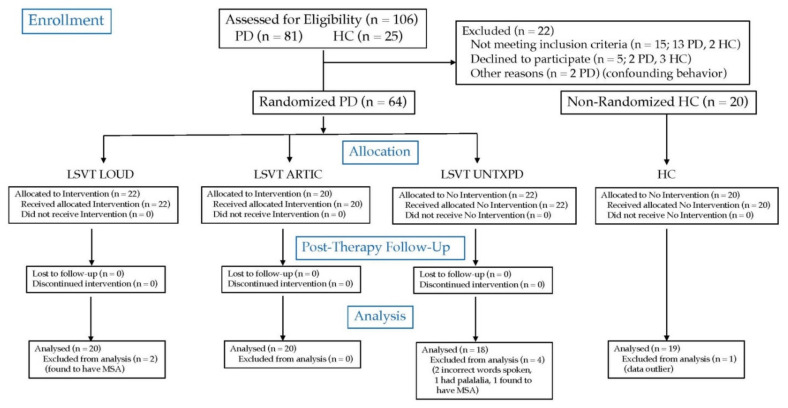
CONSORT diagram outlining the flow of participants through the trial. PD: Parkinson’s disease; HC: Healthy Control.

Participants with PD were recruited from outpatient clinics, support groups, and physicians. A total of 58 participants with PD (aged 48–85 years) were included in the analysis (see Figure 1 for the flow of participants through the trial). All PD participants were diagnosed by a neurologist, clinically stable on their antiparkinsonian medication, and within Stages I–IV on the Hoehn and Yahr scale [109]. PD participants were excluded if they: had received intensive speech treatment within the prior two years or had ever received LSVT LOUD or if they had depression (BDI ≥ 25) [110], moderate to severe dementia (MMSE ≤ 24/30) [111], a neurological condition unrelated to PD, neurosurgical treatment, vocal fold pathology (diagnosed by an ENT), or any speech or voice disorder unrelated to PD (see Appendix D for further details).

Participants with PD were randomized into three groups. The final analyses were made on 20 in the group that received intensive voice treatment (5 female, 15 male), 20 that received intensive articulation treatment (5 female, 15 male), and 18 that were in the UNTXPD group (7 female, 11 male). Participants in the UNTXPD group did not receive interventions during the study. UNTXPD participants were informed that after study completion, they could receive treatment free of charge. All participants were compensated for travel and their time.

There were no statistically significant differences (Kruskal–Wallis Test) between the three groups for any of the descriptive measures pre-treatment (see Table 1); age (*p* > 0.4039), years since diagnosis (*p* > 0.9299), Hoehn and Yahr (*p* > 0.9966), MMSE (*p* > 0.7849), BDI (*p* > 0.2044), glottal incompetence (*p* > 0.2206), swallow (*p* > 0.3362), articulation (*p* > 0.3594), and voice (*p* > 0.8481). In addition, comparison of mean DRT scores (one-way analysis of variance (ANOVA)) among the three PD groups (intensive voice treatment, intensive articulation treatment, and UNTXPD) at baseline (prior to treatment) revealed no significant difference regarding mean DRT score (F(2, 55) = 0.47, *p* = 0.63) (see Table 2). Comparison of mean DRT scores (one-way ANOVA) among the three PD groups (intensive voice treatment, intensive articulation treatment, and UNTXPD) revealed no significant differences pre-treatment in mean DRT scores for the background mall noise condition (F(2, 55) = 0.28, *p* = 0.76) and no significant differences pre-treatment in the background babble noise condition (F(2, 55) = 0.48, *p* = 0.62) (see Table 2). Thus, the PD groups were equivalent on all key variables prior to treatment.

#### 2.2.2. Healthy Control Participants

HCs were recruited through senior centers and service organizations. All analyzed HC participants (19; 12 male and 7 female, aged 46–75 years) were eligible if they had normal hearing for their age and had not smoked within the prior four years. HCs were excluded if they had depression (BDI ≥ 25) [110], moderate to severe dementia (MMSE ≤ 24/30) [111], vocal fold pathology (diagnosed by an ENT), or any speech or voice disorder (see Table 1 and Appendix D for further details). The HCs were used as a comparison group in the pre-treatment condition only due to the ceiling effect that can occur when intelligibility of PD speech in quiet environments is tested [5,78,86,99,112,113]. Pre-treatment, single word intelligibility (DRT) (with and without noise) was significantly better for HCs than the PD groups.

The study was approved by Institutional Review Boards at the University of Colorado Boulder and the University of Colorado Health Science Center with written informed consent obtained from all participants. All procedures for de-identifying shared data were followed. All participants were part of a larger ongoing research project (ClinicalTrials.gov Identifier: NCT00123084) and further descriptions of recruitment, randomization, inclusion and exclusion criteria, and randomization procedures are detailed in Ramig, et al. [62].

### 2.3. Treatments and Clinicians

Intensive voice treatment and intensive articulation treatment are Parkinson-specific neuroplasticity-principled standardized exercise-based protocols, matched on all key variables (intensity, amplitude rescaling, sensory retraining) and differing only in treatment target. As can be seen in Table 3, voice treatment has a prosodic focus, specifically, vocal loudness, whereas articulation treatment has an articulatory focus, specifically, enunciation. While the major focus of voice treatment is vocal loudness, this treatment also trains another aspect of prosody, namely, loudness across a maximum pitch range.

With both intensive voice and intensive articulation treatments, we are aiming to increase amplitude/effort to target hypokinesia; in intensive voice treatment, increased movement amplitude is directed predominately to respiratory–laryngeal systems, whereas in intensive articulation treatment, increased movement amplitude is directed predominately to the orofacial–articulatory systems (see Table 3). If there is a greater amplitude of movement, there is also an increased ROM. Additionally, by putting more effort to the articulators during reading and conversation, one is working to increase range of motion in the movement of the tongue, lips, and jaw in a functional manner. Also, more specifically, for daily task two, we used /t-k/, /n-g/, /u-i/, /u-a/ exercises (see Table 3), which more specifically target ROM.

Speech treatments were administered by three speech clinicians specializing in treating PD and certified in LSVT LOUD treatment delivery. All clinicians delivered both treatments. The principal investigators and these clinicians developed and extensively piloted intensive articulation treatment [114,115,116]. Clinicians followed established protocols for both treatments, provided the same encouragement and positive reinforcement during treatment, and conferred frequently to ensure treatment fidelity. All clinicians were compliant with IRB requirements and trained according to the University of Colorado’s required standards of clinical research.

The clinicians who delivered the treatments could not be blinded and participants were aware that they were receiving one of two treatments; however, specific treatment names (LSVT LOUD, LSVT ARTIC) were never disclosed to the participants.

Clinicians were made aware that they could impart bias in this unblinded trial and focused their effort to deliver treatments with equipoise [117] and reported that they were equally invested in both treatments.

At the end of the study, participants were asked, “out of all the treatment groups you could have been randomized into, do you feel you had the best treatment?” [118]. Positive responses were comparable between groups (100% vs. 95%, respectively). The finding that participants in both treatment groups perceived they received the most effective treatment supports that treatment delivery was similar across the two approaches and that related attempts to minimize bias were successful.

Additional details of the training of treating clinicians, control of bias, and maintaining treatment fidelity are summarized in Ramig et al. [62] and Levy et al. [75].

### 2.4. Outcomes

The primary outcome measure used to assess word intelligibility was mean percent correct on the diagnostic rhyme test (DRT) [105,106]. The DRT is a closed set (two word) selection test of 96 rhyming word pairs in which the initial consonant of each word pair differs based upon one of six distinctive perceptual features as defined by Jakobson, Fant, and Halle (compactness, graveness, sibilation, sustention, nasality, or voicing) [108]. One half of the 96 DRT word pairs (48 word pairs) were used for this study. Eight word pairs were selected for each distinctive feature, making sure to keep the vowel quadrant balanced. (see Appendix A for a consonant taxonomy used in the DRT, Appendix B for a complete list of DRT word pairs used by distinctive features, and Appendix C for the definitions of each distinctive feature). This test is effective in controlling various factors, including the amount of speaker and listener training and phonetic context, and is known to give stable intelligibility scores [106,119]. The secondary outcome was sound pressure level (SPL), an objective, acoustic measure with established reliability in studies of PD [10].

### 2.5. Data Collection and Analysis

Speech data were collected at the National Center for Voice and Speech—Denver (NCVS), an affiliate of the University of Colorado—Boulder. Additional screening/inclusion and demographic data were collected from neurology offices in Denver and the radiology department of the University of Colorado Health Sciences Center—Denver.

### 2.6. DRT

Data were collected at baseline and one month for all groups (intensive voice treatment, intensive articulation treatment, UNTXPD, and HC). One half of the 96 DRT word pairs, 48 word pairs, (see Appendix B) were presented to all participants at NCVS during pre- and post-data collection sessions. The work load would be significant for the PD participants to record a full DRT versus a half DRT. Furthermore, Dynastat studied previous DRT results from participants with PD and concluded that a half DRT is equivalent to the full list. Thus, the “half” DRT was used for this project. Words appeared one at a time on a computer screen and participants were asked to read each word when it appeared. Words were automatically presented every two seconds. Data were collected in an IAC sound-treated booth using a head-mounted AKG 420 condenser microphone positioned 8 cm from the lips. The microphone was calibrated to a Type I Sound Level Meter (Bruel and Kjaer 2238) [120] to extract dB SPL.

Early in data collection, if a participant misread or mispronounced a word, the out-of-booth examiner would immediately cycle back 2–3 words before the error and the participant would re-read the words. Later in data collection, errors were noted and words were repeated at the end (along with 2–3 words that had been read correctly so the participant could fall into their natural rhythm/voice before saying the target word).

After data collection, DRT sound files were edited at NCVS. Words were spaced to exactly two seconds apart (from beginning of one word to beginning of the next word); background noise, clicks, and pops were removed as much as possible without degrading the integrity of the word. Files were run through the AKG filter (which accounts for signal change due to the microphone). They were then reduced to 16k, and SPL measurements were taken and added to the end of the file.

Dynastat then added background noise (babble and mall) to each DRT sound file. Using standard procedures, Dynastat generated the noise. The babble noise was made of continuous speech from 30 multiple talkers (15 male and 15 female) and sampled at 16k. The mall noise was recorded in a local mall food court using a 16k sampling rate. Mall and babble noise were presented at a 0 dB SNR pre-treatment. The dB SPL of the noise file was the same both pre and post.

Dynastat assembled panels of 7–8 trained listeners. Listeners were presented with pre (mall, babble and no-noise) and post (mall, babble and no-noise) files from participants in batches. Although different batches of DRT files were presented to different listener panels, each participant had the same number of listeners pre- and post-treatment and across all noise conditions (mall, babble, no-noise). The audio files were randomized by listener panel; the audio files were not randomized for each listener. All listeners in a listener panel heard the same order during a listening session. The participants (speaker) order/treatment were presented in a counter balanced design. All listeners were blinded to the treatment and to the group membership of the participant speaker. All listeners wore Sennheiser HD25 headphones while listening to the audio files. More information can be found at http://www.dynastat.com/, accessed on 24 June 2021. As each word was played, both words in the matched pair appeared on the screen and the listener was asked to indicate which word they heard by pressing one of two buttons. Each of the files was then scored; it received a score for each of the six features and then the total score, which was the average of the feature scores. The DRT results were corrected for guessing; given a two-choice test the formula was (the number correct minus the number incorrect) divided by the number of total items. Dynastat’s listening panel members typically range in age from 18–35 years. Although there is some variation over time, no more than 60% of a panel is of one sex or the other. In order to become a member of a Dynastat listening panel, a recruit needs to meet a DRT criteria of 80% on a set of eight single speakers’ total DRT word list after three days of training. The set includes clean, low pass filtered, high pass filtered, various speech to noise ratios, and narrow band speech codec test conditions.

### 2.7. SPL

SPL data were collected at baseline and one month for each DRT word list for all groups. The cleaned (e.g., edited of coughs), calibrated microphone signals were submitted to SPL analysis using a fully automated, custom built software program designed to emulate a Type I SLM resulting in a mean and standard deviation (SD) value for dB SPL at a reference distance of 30 cm. SPL for the DRT word lists was then averaged for each PD group pre- and post-treatment and at baseline and one month for the UNTXPD group.

### 2.8. Data Sharing Statement

De-identified participant data may be available from the corresponding author by request.

### 2.9. Statistical Analysis

All statistical analyses were two-tailed and performed using statistic software (SAS, Version 9.4, Cary, NC, USA). Descriptive statistics for SPL in single words and overall DRT score pre- and post-treatment by group and listening condition are presented in Table 2 and Table 4 respectively.

For Hypothesis 1 (difference in SPL in single words for treated PD participants compared to UNTXPD):

The change in SPL from pre- to post-treatment within groups was tested using the Wilcoxon signed rank tests given slight deviations from normality. Because deviations from normality for changes were slight, and because non-parametric tests do not provide pairwise comparisons, Analysis of variance (ANOVA) was used to compare changes in SPL from pre- to post-treatment across groups with the Tukey Studentized Range test for pairwise differences (which controls the Type I experiment wise error rate).

For Hypothesis 2 (difference in single word intelligibility PD pre-treatment compared to HCs):

The difference in mean DRT score between the HC and the combined PD groups pre-treatment in each noise condition (no-noise, babble noise and mall noise) was compared using the t-test for independent samples with the Satterthwaite adjustment for unequal variances (given the test for unequal variances was significant).

For Hypothesis 3 (the effect of treatment on word intelligibility in PD in background noise):

Within PD group change in DRT scores from pre- to post-treatment were assessed using the Wilcoxon signed rank tests due to slight non-normality in distributions of differences. Because deviations from normality for changes were slight, and because non-parametric tests do not provide pairwise comparisons, Analysis of variance (ANOVA) was used to compare changes in DRT from pre- to post-treatment across groups with Tukey Studentized Range test for pairwise differences (which controls the Type I experiment wise error rate).

For Hypothesis 4 (the relationship between SPL and intelligibility):

Spearman correlations across all three PD groups and among PD groups were used to assess the relationship between changes from pre to post DRT scores and SPL with Tukey Studentized Range test for pairwise differences (which controls the Type I experiment wise error rate).

## 3. Results

Hypothesis 1 Sound Pressure Level (Difference in single word SPL for treated PD participants compared to UNTXPD):

The difference in SPL from pre- to post-treatment within groups (see Table 4) was tested using the univariate signed ranks test. Results demonstrated that only the intensive voice treatment group had significantly greater SPL post-treatment (S = 103.5, *p* < 0.0001). There were no significant differences in SPL following therapy for the intensive articulation group (S = 47, *p* = 0.06) or the UNTXPD group (S = −5.5, *p* = 0.82), although the SPL for the intensive articulation group did increase post-treatment.

Analysis of variance (ANOVA) demonstrated a main effect between groups in SPL ((F(3, 55) = 30.98, *p* ≤ 0.0001). Post-hoc analysis using Tukey’s Studentized Range (HSD) Test demonstrated that changes in SPL from pre- to post-treatment for the intensive voice treatment group were significantly greater than those for both the intensive articulation treatment (*p* ≤ 0.05) and the UNTXPD (*p* ≤ 0.05) groups.

These results confirm Hypothesis 1 that both treatment groups (intensive voice treatment and intensive articulation treatment) would demonstrate an increase in SPL following treatment but that only the intensive voice treatment group would demonstrate significantly greater SPL gains post-treatment than the intensive articulation and UNTXPD groups.

Hypothesis 2 (Difference in single word intelligibility for PD participants pre-treatment compared to HCs):

2a. In quiet environments

The difference in mean DRT scores for the HC and combined PD groups (intensive voice treatment and intensive articulation treatment, and UNTXPD) at baseline were compared using the t-test for independent samples with the Satterthwaite test. The mean DRT scores were significantly different (t(66.60) = 2.63, *p* = 0.0105) between the HC group (M = 96.67, SD = 1.45) and the combined PD groups (M = 95.25, SD = 3.19). (See Table 2).

These results confirm Hypothesis 2a. that HCs would be significantly more intelligible than the combined PD groups in the no-noise condition pre-treatment.

2b. In the presence of background noise

The difference in mean DRT score between the HC and the combined PD groups pre-treatment in the presence of noise was analyzed using the t-test for independent samples using the Sattertwaite test. In the presence of background mall noise, the HC group mean DRT score (82.3/5.3) was significantly greater than the combined PD groups mean DRT score (72.1/17.9) (t = 4.47 (74.19), *p* < 0.0001). Likewise, in the presence of background babble noise, the HC group mean DRT score (88.1/4.4) was significantly greater than the combined PD groups mean DRT score (80.2/13.1) (t = 4.53 (70.94), *p* < 0.0001).

These results confirm Hypothesis 2b., that the HC group would be significantly more intelligible than the combined PD groups in the presence of background mall and babble noise pre-treatment.

Hypothesis 3 (Effect of treatment on word intelligibility in PD in background noise):

3a. Difference between treated PD groups and UNTXPD

Within PD group, change in DRT scores pre- to post-treatment was assessed using the Wilcoxon signed rank tests. In both the mall and babble noise conditions, mean DRT scores were significantly higher post treatment for the intensive voice treatment group (S = 84.5, *p* = 0.0007; S = 92, *p* = 0.0002, respectively) and intensive articulation group (S = 58, *p* = 0.02; S = 58, *p* = 0.03, respectively) but the change in mean DRT score from pre- to post- for UNTXPD group was not significant (S = −14.5, *p* = 0.54; S = −2.5, *p* = 0.82, respectively).

This result confirms Hypothesis 3a., that both PD treatment groups would demonstrate a significant increase in intelligibility (higher DRT score) compared to the untreated PD group from pre- to post-treatment.

3b. Differences Among PD groups

In the mall noise condition, a mixed effects model showed a significant difference in trend from pre- to post-treatment among groups regarding mean DRT score (F(2, 55) = 5.98, *p* = 0.0045). Post-hoc analyses indicated that the mean DRT scores for the treatment groups were significantly greater post-treatment (*p* < 0.05), but there was no significant change for UNTXPD group (*p* = 0.5920). Differences among groups in mean DRT scores post-treatment were not significantly different (*p* > 0.05). There was a significant difference among groups in mean DRT score in the babble noise condition (F(2, 55) = 7.61, *p* = 0.0012). Post-hoc analyses indicated that the mean DRT score for the intensive voice treatment group was significantly greater post-treatment than the mean DRT scores for UNTXPD and for the intensive articulation group (adjusted *p*-value < 0.05).

This result partially confirms Hypothesis 3b., that in the presence of background Mall noise, the intensive voice treatment group was significantly more intelligible post-treatment than the UNTXPD group but not the intensive articulation group. However, in the presence of background babble noise, the intensive voice treatment group was significantly more intelligible post-treatment than both the intensive articulation treatment group and the UNTXPD groups.

Hypothesis 4 (Relationship between SPL and word intelligibility):

Correlations (Spearman) across all three PD groups of changes from pre- to post-treatment DRT scores and SPL were significant and positive for each of the three listening conditions no-noise (r = 0.27, *p* = 0.04; weak but significant correlation), mall noise (r = 0.66, *p* < 0.0001; moderate–strong and significant correlation), and babble noise (r = 0.70, *p* < 0.0001; strong and significant correlation).

Within group correlations for changes from pre- to post-treatment between DRT and SPL for the no-noise condition were not significant (intensive voice treatment r = 0.20, *p* = 0.39; intensive articulation treatment r = 0.28, *p* = 0.23; UNTXPD r = 0.43, *p* = 0.07).

For the mall noise condition, correlations for changes from pre- to post-treatment between DRT and SPL for each of the three PD groups were moderate and positive (intensive voice treatment: r = 0.50, *p* = 0.03; intensive articulation treatment: r = 0.58, *p* = 0.0071; UNTXPD: r = 0.55, *p* = 0.02).

For the babble noise condition, correlations for changes from pre- to post-treatment between DRT and SPL for each of the three PD groups were moderate and positive (intensive voice treatment: r = 0.49, *p* = 0.03; intensive articulation treatment: r = 0.51, *p* = 0.02; UNTXPD: r = 0.58, *p* = 0.01).

These results confirm Hypothesis 4, that there would be a significant relationship between loudness (SPL) and intelligibility (mean DRT score) such that as SPL increased, mean DRT scores would also increase, especially in the two noise conditions.

## 4. Discussion

Previous RCT studies comparing intensive voice treatment to intensive articulation treatment have shown improvements in vocal loudness, communication effectiveness, and self-generated sentence intelligibility in background noise [62,75]. The current study is the first RCT study to examine word intelligibility in PD following two intensive treatments, one targeting the prosodic feature of vocal loudness, intensive voice treatment, and one targeting articulation, intensive articulation treatment, in the presence of two different background noise conditions. The DRT methodology used in this study systematically inventories a comprehensive range of vocal tract valving. Overall, the results indicated that while both treatment groups demonstrated an increase in word intelligibility compared to the untreated PD group post-treatment, the treatment targeting the prosodic feature of vocal loudness improved word intelligibility to a greater degree than treatment targeting articulation. The discussion will first consider the results of the SPL analysis (Hypothesis 1), then the pre-treatment (baseline) results (Hypotheses 2), post-treatment results (Hypothesis 3), and finally, the relationship between loudness and intelligibility (Hypothesis 4).

### 4.1. Sound Pressure Level: Do Treated PD Participants Have Greater Gains in SPL for Single Words Than Untreated PD Participants Following Treatment?

As hypothesized, and in agreement with previous research [62], both treatment groups demonstrated an increase in SPL following treatment; however, only the intensive voice treatment group demonstrated greater SPL gains post-treatment than the intensive articulation treatment and UNTXPD groups. This is the first report of gains in loudness in single words following intensive voice treatment. These results also are in agreement with previous research [62] comparing sentence level increases in SPL following intensive voice and articulation treatment.

### 4.2. Are HCs More Intelligible for Single Words Than PD Participants Pre-Treatment with and without Background Noise?

The results reported here comparing word intelligibility between the combined PD groups and HCs prior to treatment in the absence of background noise are in agreement with previous studies that demonstrated that the intelligibility of speech of people with PD is significantly reduced compared to healthy age-matched speakers [5,49,81]. However, although statistically significant (Table 2), the three PD groups each had single word intelligibility averaging 95% compared to HCs averaging 97%. Taken together, our results support that while single word intelligibility for people with PD can be significantly reduced compared to HCs, single word intelligibility for people with PD is relatively good in quiet listening environments. Thus, these results support the need to determine the intelligibility of speech in PD in the presence of background noise, which is not only ecologically motivated but also addresses a significant complaint of people with PD [56].

Our results confirmed that single word intelligibility in people with PD would deteriorate in the presence of background noise compared to age-matched HC speakers. The HC group demonstrated significantly greater word intelligibility (mean overall DRT score) than the combined PD groups in the presence of both background mall and babble noise prior to treatment. These results are the first to report reduced intelligibility of single words in speakers with PD in two background noise environments. They are in agreement with the results of Chiu and Forrest [49] and Leszcz [81], who found significant reductions in the intelligibility of words in participants with PD in the presence of background babble noise, as well as previous studies that have reported reduced intelligibility for sentences in speakers with PD [75,92] in background noise. These findings corroborate the complaints of people with PD having difficulty being understood in noisy environments [47]. These results are also in agreement with studies that report people with PD have difficulty maintaining intelligible speech in the presence of background noise [5,6,10,82,83,84]. In addition, these results further support the need to evaluate the intelligibility of speech in speakers with PD in the “ecologically” relevant presence of background noise. Additionally, our speakers with PD were the same as those in the Levy et al. [75] study, and therefore, our results point to the fact that if speakers with PD have reductions in single word intelligibility in background noise, they will also have reductions in intelligibility at “higher” levels of speech production, such as at the sentence level and possibly at the conversational level, as suggested by prior researchers [78,79,80]. This observation has clinical implications in that the use of single words to assess intelligibility is a much less difficult task for dysarthric participants than “higher” levels of speech production and so may be easier to collect clinically.

### 4.3. What Is the Effect of Treatment on Word Intelligibility in PD in the Ecologically Valid Situation of Background Noise?

Our results confirmed that both PD treatment groups would demonstrate an increase in word intelligibility compared to the untreated PD group post-treatment. Thus, both forms of intensive speech treatment were found to improve single word intelligibility in agreement with a previous study [75] that measured sentence intelligibility following intensive voice and articulation treatment compared to an untreated group of people with PD.

The increased intelligibility of words following intensive voice treatment can be attributed to the improvements in overall prosodic and articulatory systems following this treatment that have been well documented. Increased vocal loudness (SPL), a feature of prosody, has been shown to result in system-wide effects, such as in measures of articulation [51,53,98,101,103], speech rate [121], intonation [64], aerodynamics [65], and perceptual measures of voice quality [7], in addition to measures of speech intelligibility [75,86,89,122]. Interestingly, several studies have noted reductions in movement amplitude of the articulators [51,96,97] that corresponded to reductions in intelligibility. Two of these studies [96,97] also noted an increase in the average speed of articulatory movement for the jaw and tongue, and Kearney et al. [57] found increased jaw and tongue amplitudes and velocities for sentences of PD speakers speaking loudly. Although the PD speakers in the Kearney et al. [51] study were only cued to speak louder and did not receive intensive treatment targeting voice, it may be the case that intensive treatment targeting voice has articulatory benefits beyond those noted for increasing articulatory amplitude, and the increase in articulatory velocity may also be a contributing factor to an increase in word intelligibility.

The increased intelligibility of words following intensive articulation treatment compared to the untreated PD group supports the relationship of articulation and intelligibility noted previously [93,94]. The results of Levy et al. [75] also noted increased intelligibility for sentences following intensive articulation treatment, although the differences between the intensive articulation treatment group and the untreated group were not statistically significant.

We predicted that the treatment targeting the prosodic feature of vocal loudness would result in greater word intelligibility than both the treatment targeting articulation and the untreated PD speakers in the presence of both mall and babble background noise. This prediction was confirmed in the presence of background babble noise. The intensive voice treatment group had significantly greater word intelligibility post-treatment than both the intensive articulation treatment and the UNTXPD groups. This result is in agreement with the findings of Levy et al. [75], who demonstrated the intensive voice treatment group was significantly more intelligible for sentences (% accurately transcribed words) than the intensive articulation and untreated PD groups in background babble noise, and Cannito et al., [92] who demonstrated increased sentence intelligibility following LSVT LOUD treatment in the presence of background pink noise.

However, in the presence of background mall noise, the intensive voice treatment group had significantly greater word intelligibility post-treatment than the UNTXPD group but not than the intensive articulation group. Although not statistically significantly different, the intensive voice treatment group did have a higher overall mean DRT score than the intensive articulation treatment group in the presence of background mall noise. This difference from pre- to post-treatment (7.4%) was actually greater than the difference in overall mean DRT score between the two treatment groups (6.7%) in the background babble noise condition; however, the variability in scores was greater in the background mall noise condition. The greater variability in DRT scores in the background mall noise than the background babble noise condition could be attributed to the differences in the spectrum of the two types of noise. That is, the spectrum of the background mall noise, recorded from a local mall food court, presented a broad spectrum of noise covering the speech frequencies. In contrast, the background babble noise condition, made of continuous speech from 30 multiple talkers, only consisted of speech spectrum noise. The addition of other types of noise in the background mall noise condition may therefore have increased the variability of mean DRT scores from that condition. Additionally, when mixed with the DRT word list, different spectral characteristics of noise could be present at different times in the word list for different participants. The order of DRT word lists was randomized for all participants, so even if the mall was “stationary” the same spectral characteristics would not always be present with the same words, as the order of the word lists were different. These results support that the intensive voice treatment group had greater word intelligibility in both background babble and mall noise conditions post-treatment than the intensive articulation treatment group.

### 4.4. What Is the Relationship between Increased Loudness and Word Intelligibility?

Our results demonstrated a significant relationship between loudness and word intelligibility such that as loudness (SPL) increased, mean DRT scores also increased, especially in the two noise conditions. Thus, an increase in loudness is positively associated with an increase in word intelligibility and supports, at least in part, that an increase in the prosodic feature of vocal loudness promotes increased intelligibility more than a focus on articulation. This relationship is in line with the study by Levy et al. [75] but not with others, who noted that articulation had a greater effect on intelligibility [93,94]. In those studies, however, intelligibility was not assessed in the presence of background noise.

### 4.5. Limitations

There are some limitations of this study that are worth considering. The first is that we used a single word reading task and, thus, only considered one dimension of speech intelligibility. However, the results of this RCT add to the results of studies that assessed other aspects of PD speech intelligibility following intensive treatment targeting the prosodic feature of vocal loudness, namely, at the sentence level [75,92] and the conversational level [89,90], to provide a growing body of research demonstrating that intensive treatment targeting the prosodic feature of vocal loudness results in significant improvements in speech intelligibility for people with PD. As the conversational intelligibility of PD speech was evaluated in languages other than English [89,90], further study of conversational intelligibility of PD speech is warranted in English, as well as other languages.

Individual speaker characteristics, such as gender, voice quality, and fatigue, may also impact the perception of intelligibility. The effect of gender on intelligibility is complex. The relatively little research that has been devoted to this question in healthy speakers has come to conflicting conclusions. Some studies found female voices to be more intelligible than males [123,124,125], some found male talkers to be more intelligible than female talkers [126], and some found male and female voices to be equivocal in terms of intelligibility [127]. There is only one study that we are aware of that addresses this question in neurologically impaired speakers. That study [78] found no significant difference in single word intelligibility by gender in dysarthric participants with MS or PD nor in HCs. Females demonstrate a greater F0 variation when speaking [125], and it is known that a greater degree of F0 variation positively affects intelligibility [128,129,130]. However, a difference in F0 variability would more likely affect the intelligibility of sentences than the monosyllabic words used in the present study. In addition, in previous studies [60,61,62,75], no difference in the magnitude of treatment effects was found on the basis of the gender of the participants. Nonetheless, future studies should address the potential gender differences in treatment outcomes, including intelligibility when treatment groups are balanced by gender. Voice quality characteristics, such as hoarseness and breathiness, have been documented in speakers with PD [26] and likely were present in the speech of our PD speakers as well. Although the three PD groups in the present study were comparable pre-treatment on voice impairment, which included ratings of hoarseness and breathiness (see Table 1 and Appendix D), individual PD speaker voice quality may have impacted ratings of word intelligibility. Individual speaker fatigue may also have impacted ratings of word intelligibility given the long list of words the PD speakers had to read. If a PD speaker were more fatigued pre- versus post-treatment or vice versa, their word intelligibility scores might, thus, be affected.

Finally, neither clinicians providing treatment nor participants could be blinded because this is a behavioral intervention trial. However, great care was taken to evaluate reliability, ensure equipoise, implement standardized training, minimize bias in data collection and analysis, and maintain independence between treating clinicians and those recording data. The finding that participants in both treatment groups perceived they received the most effective treatment supports that treatment delivery was similar across the two approaches and that related attempts to minimize bias were successful.

### 4.6. Future Directions

Although vowel changes have been noted that correlate with improved PD speech intelligibility [18,98,99,100,101,102,103], changes in the articulation of consonants that may contribute to improved intelligibility have been less well documented [55,86]. These findings warrant further investigation to determine what characteristics of consonants may contribute the most to word intelligibility. Future research will assess individual DRT features to document specific types of consonant changes pre- to post-treatment. The single word methodology of the DRT uniquely facilitates this valuable form of analysis.

## 5. Conclusions

Approximately 90% of people with PD have prosodic and articulatory signs, including reduced vocal loudness and difficulty with articulation. These changes in communication lead to a reduction in speech intelligibility, which has been reported to contribute to significant declines in functional communication, communicative participation, and quality of life. Speech intelligibility, the extent to which others can understand speech, is negatively affected by a reduction in audibility and imprecise articulation. Our results confirm that single word intelligibility in quiet listening environments, though relatively good, is reduced in speakers with PD compared to healthy age-matched controls. In addition, difficulties being understood are exacerbated when people with PD speak in the presence of background noise. Our PD participants had significantly reduced single word intelligibility in both background noise conditions compared to healthy age-matched controls. The background noise most commonly encountered in everyday life is speaking in a group of people who are also speaking; i.e., background babble noise. The results of this RCT, in conjunction with those of Levy et al. [75], demonstrated that the intelligibility of speech at the single word and sentence level in the presence of background babble noise was significantly improved after intensive voice treatment. In contrast, the intensive articulation treatment group did not demonstrate significantly greater single word intelligibility post-treatment in background babble noise. In addition, our results also demonstrated that the intelligibility of words in the presence of background mall noise was greater for the group that received intensive speech treatment targeting the prosodic feature of vocal loudness than the group that received intensive speech treatment targeting articulation. Thus, intensive prosodic treatment targeting vocal loudness has a greater impact on improving the intelligibility of speech than intensive treatment targeting articulation. When added to the many other studies documenting improvement in various aspects of speech following intensive voice treatment, these findings demonstrate that the prosodic target of vocal loudness has a positive effect on improving intelligibility in PD speakers. Furthermore, these data contribute to the advances in rehabilitation in PD, which both improve quality of life while advancing our understanding of the underlying physiology and neural bases [71,72,73] supporting these changes.

## Figures and Tables

**Table 1:** Participant Descriptive Statistics.

Descriptive Variables and Demographics	LSVT LOUD (*N* = 20)	LSVT ARTIC (*N* = 20)	UNTXPD (*N* = 18)	HC (*N* = 19)
Gender				
Male	15	15	11	12
Female	5	5	7	7
**Variables mean (range)**				
Age in years	67 (49–85)	68.2 (53–85)	63.5 (48–81)	63.3 (46–75)
Years Since Diagnosis	4.89 (0.07–31)	5.10 (0–20)	4.64 (0.5–14)	--
Hoehn and Yahr Stage with medication	2.13 (1–3)	2.23 (1–4)	2.14 (1–3)	--
MMSE	28.8 (26–30)	28.7 (27–30)	29.1 (28–30)	29.4 (27–30)
BDI	9.6 (1–20)	9.05 (0–20)	6.72 (1–15)	2.89 (0–13)
Glottal Incompetence	1.75 (0–3)	2.45 (0–4)	1.94 (0–4)	1.53 (0–3)
Swallow	1.25 (0–3)	1.15 (0–3)	0.83 (0–2)	0.26 (0–2)
Articulation	0.70 (0–3)	0.80 (0–2)	0.56 (0–2)	0.05 (0–1)
Voice	1.70 (1–3)	1.75 (1–3)	1.61 (1–3)	0.63 (0–2)

Note. Means and ranges for descriptive measures pre-treatment. Voice and articulation were measured on a scale from 0–5, where 0 = no disorder and 5 = severe disorder. HC = Healthy Controls; MMSE = Mini-Mental State Examination; BDI = Beck Depression Index. See Appendix D for further information on screening and inclusion/exclusion criteria.

**Table 2:** Comparison of LSVT LOUD^®^ and LSVT ARTIC^TM^ speech therapy for PD.

	LSVT LOUD	LSVT ARTIC
Focus of Treatment	Loudness	Enunciation
**Dosage**	Increased movement amplitude directed predominately to respiratory–laryngeal systems	Increased movement amplitude directed predominately to orofacial–articulatory system
	Individual treatment session of one hour, four consecutive days per week over a 4-week period	Individual treatment session of one hour, four consecutive days per week over a 4-week period
**Effort**	Push for maximum participant perceived effort	Push for maximum participant perceived effort
**Daily Exercises**		
Maximum sustained activities completing multiple repetitions of tasks (min. 1–12)	Sustain the vowel “ah” in good quality, louder voice, as long as possible	Sustain articulatory placement for “p” (lips closed) “t” (tongue tip behind upper teeth) with Iowa oral pressure instrument (IOPI) for a 4-s hold
Range activities completing multiple repetitions of tasks (min. 13–23)	Say the vowel “ah” in loud good quality voice going high in pitch; hold for 5 s	Repeat as many as possible, in 5 s trials, each of the following single consonants with precise articulation (voiceless productions): /p/ /t/ /k/
	Say the vowel “ah” in loud good quality voice going low in pitch; hold for 5 s	Repeat as many as possible, in 5 s trials, each of the following minimal pair combinations with precise articulation: /t-k/, /n-g/, “oo-ee” and “oo-ah”
Functional activities (min. 24–30)	Participant reads 10 self-generated phrases he/she says daily in functional living (e.g., “good morning”) using the same effort and loudness as he/she did during the maximum sustained and range exercises	Participant reads 10 self-generated phrases he/she says daily in functional living (e.g., “good morning”) using same effort for enunciation as he/she did during the maximum sustained and range exercises.
Hierarchy Exercises (min. 31–55)		
**Purpose**	Train rescaled vocal loudness and pitch range achieved in the daily exercises into context specific and variable speaking activities	Train rescaled enunciation achieved in the daily exercises into context specific and variable speaking activities
**Method**	Incorporate multiple repetitions of reading and conversation tasks with a focus on vocal loudness	Incorporate multiple repetitions of reading and conversation tasks with a focus on enunciation
**Tasks**	Tasks increase in length of utterance and difficulty across weeks, progressing from words to phrases to sentences to reading to conversation, and can be tailored to each participant’s goals and interests (e.g., golf vs. cooking)	Tasks increase in length of utterance and difficulty across weeks, progressing from words to phrases to sentences to reading to conversation, and can be tailored to each participant’s goals and interests (e.g., golf vs. cooking)
**Assign Homework Exercises** (min. 56–60)	Subset of the daily exercises and hierarchy exercises to be completed outside of the therapy room	Subset of the daily exercises and hierarchy exercises to be completed outside of the therapy room
Assignment	Participant is to use the louder voice practiced in exercises in a real-world communication situation	Participant is to use enunciated speech practiced in exercises in a real-world communication situation
Difficulty level	Matched to the level of the hierarchy where the participant is in treatment	Matched to the level of the hierarchy where the participant is in treatment
Duration and repetitions on treatment days (4 days/week)	10 min, performed once per day	10 min, performed once per day
**Shaping techniques**		
Purpose and approach	Train vocal loudness that is healthy (i.e., no unwanted vocal strain) through use of modeling (“do what I do”) or tactile/visual cues	Train speech enunciation that is within normal limits (i.e., no excessive movements) through use of modeling (“do what I do”) or tactile/visual cues
**Sensory calibration treatment**	Focus attention on how it feels and sounds to talk with increased vocal loudness	Focus attention on how it feels and sounds to talk with increased enunciation
**Objective and subjective clinical data collected during each treatment session**	Measures of duration, frequency, and sound pressure level	Measures of oral pressure and precise articulatory productions
	Documentation of percentage of cueing required to implement vocal loudness strategy	Documentation of percentage of cueing required to implement enunciation strategy
	Observations of perceptual voice quality	Observations of perceptual speech intelligibility
	Participant self-reported comments about successful use of the improved loudness in daily communication	Participant self-reported comments about successful use of the improved enunciation in daily communication
	Participant self-reported perceived effort	Participant self-reported perceived effort

Note. Both therapies are standardized with respect to intensive dosage. Effort in LSVT LOUD and LSVT ARTIC are based on the patient’s self-perceived effort during treatment tasks, on a scale of 1–10, with 10 being highest perceived effort.

**Table 3:** Descriptive statistics for SPL (dB at 30cm) pre-treatment and 1 month post-treatment by group.

		SPL	
		PRE	POST
Group	*N*	Mean (SD)	Mean (SD)
LSVT LOUD	20	73.4 (3.80)	79.4 (3.85)
LSVT ARTIC	20	73.6 (3.27)	74.6 (3.11)
UNTXPD	18	72.1 (4.19)	71.9 (3.66)
HC	19	73.3 (3.45)	73.4 (3.61)

Note. SPL = sound pressure level; UNTXPD = Untreated PD, HC = healthy controls.

**Table 4:** Mean DRT (sd) by group in each listening condition.

	NO NOISE		MALL NOISE		BABBLE NOISE	
	PRE	POST	PRE	POST	PRE	POST
LSVT LOUD	95.0 (3.9)	96.1 (3.6)	72.2 (16.3)	84.6 (10.8)	78.7 (12.6)	89.7 (6.7)
LSVT ARTIC	95.8 (2.8)	95.0 (3.5)	70.3 (14.2)	77.2 (11.0)	80.0 (10.3)	83.0 (10.0)
UNTXPD	94.9 (2.8)	96.0 (1.8)	73.9 (13.7)	72.3 (15.7)	82.1 (9.1)	80.5 (13.5)
HC	96.7 (1.4)	96.8 (1.43)	82.3 (5.3)	81.4 (9.6)	88.1 (4.4)	87.7 (4.8)

## Data Availability

The de-identified participant data presented in this study are available on request from the corresponding author.

## Appendix A

**Table A1:** Consonant Taxonomy used in construction of the DRT.

	PHONEMES																						
Features	[ m	n	v	ð	z	ʒ	$\hat{ʒ}$	b	D	g	w	r	l	j	f	θ	s	ʃ	$\hat{ʃ}$	p	t	k	h ]
Voicing	+	+	+	+	+	+	+	+	+	+	+	+	+	+	−	−	−	−	−	−	−	−	−
Nasality	+	+	−	−	−	−	−	−	−	−	−	−	−	−	−	−	−	−	−	−	−	−	−
Sustention	−	−	+	+	+	+	−	−	−	−	+	+	+	+	+	+	+	+	−	−	−	−	+
Sibilation	−	−	−	−	+	+	+	−	−	−	−	−	−	−	−	−	+	+	+	−	−	−	−
Graveness	+	−	+	−	−	∅	∅	+	−	∅	+	−	∅	∅	+	−	−	∅	∅	+	−	∅	∅
Compactness	−	−	−	−	−	+	+	−	−	+	−	−	∅	+	−	−	−	+	+	−	−	+	+

*Key:* + = present; − = absent; ∅ = does not apply.

## Appendix B

**Table A2:** Diagnostic Rhyme Test Word Pairs by Distinctive Feature Category ^a,b^.

VOICING	NASALITY	SUSTENSION
*Voiced—Unvoiced*	*Nasal—Oral*	*Sustained—Interrupted*
gin—chin	mitt—bit	vee—bee
dint—tint	nip—dip	sheet—cheat
voal—foal	moot—boot	foo—pooh
goat—coat	news—dues	those—doze
zed—said	mend—bend	then—den
dense—tense	neck—deck	fence—pence
vault—fault	moss—boss	shaw—chaw
daunt—taunt	gnaw—daw	vox—box
**SIBILATION**	**GRAVENESS**	**COMPACTNESS**
*Sibilated—Unsibilated*	*Grave—Acute*	*Compact—Diffuse*
zee—thee	weed—reed	yield—wield
cheep—keep	peak—teak	key—tea
jab—gab	bank—dank	shag—sag
sank—thank	fad—thad	gat—bat
juice—goose	moon—noon	ghost—boast
chew—coo	pool—tool	show—so
jaws—gauze	wad—rod	got—dot
saw—thaw	pot—tot	hop—fop

^a^ Voiers ([106], p. 16). ^b^ Dynastat.

## Appendix C

**Table A3:** Definitions of Distinctive Feature Categories ^a,b^.

Term	Definition
Compactness	The essential articulatory difference between the compact and diffuse phonemes lies in the relation between the volume of the resonating cavities in front of the narrowest stricture and those behind this stricture. The ratio of the former to the latter is higher for the compact than for the corresponding diffuse phonemes. Hence, consonants articulated against the hard or soft palate (velars and palatals) are more compact than the consonants articulated in the front part of the mouth.
Graveness	The gravity of a consonant is generated by a larger and less comparted mouth cavity, while acuteness originates in a smaller and more divided cavity. Hence, gravity characterizes labial consonants as against dentals, as well as velars vs. palatals.
Sibilation	Corresponds to the strident–mellow classification of Jakobson, Fant, and Halle (1952). Sibilant consonants are primarily characterized by a noise, which is due to turbulence at the point of articulation.
Sustention	Corresponds to the continuant vs interrupted classification of Jakobson, Fant, and Halle (1952). Classifies consonants into clearly continuous consonants and other transient phones, such as plosives.
Nasality	The oral (or more exactly, the non-nasalized) phonemes are formed by the air stream, which escapes from the larynx through the mouth cavity only. The nasal (or more exactly, nasalized) phonemes are, on the contrary, produced with a lowering of the soft palate, so that the air stream is bifurcated and the mouth resonator is supplemented by the nasal cavity.
Voicing	Voiced phonemes are emitted with concomitant periodic vibrations of the vocal folds and voiceless phonemes without such vibrations.

^a^ Jakobson, Fant, and Halle (pp. 23–30, 39–40, [108]). ^b^ Kondo ([119], p. 157).

## Appendix D

A. *Exclusion Criteria for Participants with Parkinson’s Disease (PD) and Healthy Controls (HC)*

Primary inclusion and exclusion criteria for participants with PD are summarized in the Methods. Additional exclusion criteria are listed here: drug abuse, significant history of gastrointestinal disease or surgery, head or neck cancer, severe temporomandibular joint disorder, or pregnancy (or the possibility of pregnancy) specific to the modified barium swallow study (MBS).

Exclusion criteria specific to HC participants are listed here: being in generally poor health, history or current complaints of: voice or speech disorder, neurological condition, learning disability or psychiatric condition, or pregnancy (or the possibility of pregnancy, due to MBS study).

B. *Screening for Inclusion*

Phase 1: Voice, speech, hearing, depression, and cognition. The voice and speech screening, comprised of phonation, reading, and speaking tasks, was administered by speech clinicians expert in assessing and treating voice and speech in PD. Two clinicians rated the participants’ voice and speech during this session, based on the criteria outlined in the form below. During the same session, a hearing screening was conducted and depression (Beck depression inventory (BDI-II)) and cognition (mini mental status exam (MMSE)) scales were administered.

**Table A4:** Study: Efficacy of Speech Treatment in Parkinson’s Disease.

Subject:_______________	Date:___________		Examiner:_______________
DOMAIN			
**Articulation**	*Phonation*	*Reading* *Grandfather*	*Conversation* *Family* *1 min*
Imprecise consonants			
Variable rate			
Slow/fast rate			
Rushes			
Other			
**Artic severity** **(normal, mild, mod, severe)**			
**Voice**			
Reduced SPL			
Reduced variability			
High/Low F0			
Breathy			
Hoarse			
Strained/Hyperfunction			
Other			
**Voice severity** **(normal, mild, mod, severe)**			
**Resonance**			
Hypernasal			
Hyponasal			
Emission			
**Resonance Severity** **(normal, mild, mod, severe)**			
**Total Artic Severity: ___________** **Total Voice Severity: ___________** **Total Resonance Severity: ___________** **Overall Severity Rating: ____________** **Overall % intelligible in conversation: ____________**			
Ranges: 0 = normal 0.5–2 = mild 2.5–3.5 = moderate 4–5 = severe			

Voice and speech severity were rated by two speech clinicians during the clinical screening visit as follows: participants were asked to perform a variety of voice and speech tasks, including sustained phonation, paragraph reading, and spontaneous conversation, which were rated independently based on voice characteristics (e.g., loudness, quality, variability), resonance, and speech articulation (e.g., consonant precision, rate). A severity scale ranging from none (0)–severe (5) was used to globally rate articulation, voice, and resonance for each of the three tasks where appropriate (e.g., phonation was not used to rate articulation). Within each severity category, clinicians chose values to reflect a further degree of specificity (0.5–2 = mild, 2.5–3.5 = moderate, 4–5 = severe). The two independent clinician ratings were then averaged for each participant to determine severity levels for voice and speech. In the event that there was disagreement that resulted in assigning severity to a different category (mild vs. moderate), the clinicians conferenced to come up with a consensus. Categories of none, mild, moderate, and severe were used in the minimization program for randomization.

Phase 2: Videolaryngoscopy exam. An otolaryngologist (ENT) MD or trained speech clinician administered the tasks outlined in the standardized protocol listed below. All exams were reviewed by the ENT for possible contraindications or conditions that would indicate a laryngeal presentation not typical of PD and, thus, preclude inclusion.

**Table A5:** Assessment of velopharyngeal function.

Evaluate:		Closure		
		Coordination		
		Tremor		
*2. Evaluation of larynx, without stroboscopy*				
Tasks:		rest breathing		
		repeat “ee-hee”		
		repeat “puh–tuh–kuh”		
		repeat “ee”-sniff		
		whistle		
		sing “happy birthday”		
		brief conversation		
**Evaluate**:		**presence (and type) of masses**		
		**glottal configuration during phonation**		
		**evidence of supraglottic hyperfunction (A–P, ventricular)**		
		**evidence/symptoms of GERD (none, mild, moderate, severe)**		
**Is GERD (Gastroesophageal Reflux) too severe that the subject should not start TX?**		**Yes/No**		
**Does the subject need a RX for reflux medication?**		**Yes/No**		
**Did ENT write a RX for reflux medication?**		**Yes/No**		
**If yes, list medication and dose___________________________________________**				
**excess mucous ____________________________________________**				
**dyskinesias _______________________________________________**				
**Determine degree of glottal incompetence (0–5)**				
1 = mild	2 = mild/mod	3 = moderate	4 = mod/severe	5 = severe
**Rigid Exam**				
*1. Evaluation of larynx, with stroboscopy*				
Tasks:	rest breathing			
	sustained “ee” at comfortable pitch and loudness			
	sustained “ee” at high pitch			
	sustained “ee” at low pitch			
	loud “ee” at comfortable pitch			
	soft “ee” at comfortable pitch			
**Evaluate:**	**fill in machine-generated strobe report**			
**Determine degree of glottal incompetence (0–5)**				
1 = mild	2 = mild/mod	3 = moderate	4 = mod/severe	5 = severe

### Other Comments:

Phase 3: Swallowing study. Modified barium swallow (MBS) studies were conducted in a radiology suite by trained speech clinicians who administered boluses in varying consistencies during videofluoroscopy according to the standardized protocol listed below.

**Table A6:** Study: Efficacy of Speech Treatment in Parkinson’s Disease.

Patient Name:_______________________________						Study ID#:______________________						
Date of Evaluation__________			Time of Day__________						Time of Last Medication____________			
**VIDEOFLUOROSCOPIC STUDY OF SWALLOWING PROTOCOL**												
**Announce each bolus on the audio channel before each swallow.**												
***Check box or circle appropriate response as bolus is presented.***												
***(NP = not presented; Asp = Aspirate; IC = intervention code)***												
	**Trial 1**		**Trial 2**			**Trial 3**			**Trial 4**			
**Lateral View**	**T 1** **Y/N**	**Asp** **Y/N**	**T 2** **Y/N**	**Asp** **Y/N**	**IC**	**T 3** **Y/N**	**Asp** **Y/N**	**IC**	**T 4** **Y/N**	**Asp** **Y/N**	**IC**	**Bolus not presented** **(explain why)**
1 mL liquid *												
3 mL liquid *												
5 mL liquid **												
10 mL liquid **												
1 swallow of liquid/cup												
3 mL pudding												
1/4 Lorna Doone cookie with barium on top												
**Anterior/Posterior View**	**T 1**	**Asp** **Y/N**	**T 2**	**Asp** **Y/N**	**IC**	**T 3**	**Asp** **Y/N**	**IC**	**T 4**	**Asp** **Y/N**	**IC**	**Bolus not presented** **(explain why)**
3 mL pudding												

* Given from teaspoon after measuring with syringe; ** Measured and given from syringe.

**Rehabilitative Interventions** Used during x-ray if patient aspirates:

Key Intervention Codes (IC):

1. Chin down	3. Head turned	5. Lying down	7. Other (specify
2. Chin up	4. Head tilted	6. None	

If no intervention was provided, please indicate why:

**Severity rating:** __________________________

**Normal**: No aspiration on any swallows, oropharyngeal transit times 2.0 s or less on pudding swallows; no or minimal residue on all swallows.

**Mild**: No aspiration on any swallows, oropharyngeal transit time 2–5 s on pudding swallows, and/or residue partially filling the valleculae or pyriform sinuses on any swallow.

**Moderate**: Could include any one of the following; trace aspiration, oropharyngeal transit time of 6–10 s, residue filling the valleculae or pyriform sinuses on pudding swallows.

**Severe**: Aspiration and/or oropharyngeal transit times 11 s or more on any swallow; residue filling the valleculae and pyriform sinuses on pudding swallows.
